# Structural connectivity of the sensorimotor network within the non-lesioned hemisphere of children with perinatal stroke

**DOI:** 10.1038/s41598-022-07863-4

**Published:** 2022-03-09

**Authors:** Brandon T. Craig, Eli Kinney-Lang, Alicia J. Hilderley, Helen L. Carlson, Adam Kirton

**Affiliations:** 1 Calgary Pediatric Stroke Program, Cumming School of Medicine, University of Calgary, Calgary, AB Canada; 2 Hotchkiss Brain Institute, Cumming School of Medicine, University of Calgary, Calgary, AB Canada; 3 Alberta Children’s Hospital Research Institute, Cumming School of Medicine, University of Calgary, Calgary, AB Canada; 4 Department of Pediatrics, Cumming School of Medicine, University of Calgary, Calgary, AB Canada; 5 Department of Clinical Neuroscience, Cumming School of Medicine, University of Calgary, Calgary, AB Canada

**Keywords:** Motor cortex, Stroke

## Abstract

Perinatal stroke occurs early in life and often leads to a permanent, disabling weakness to one side of the body. To test the hypothesis that non-lesioned hemisphere sensorimotor network structural connectivity in children with perinatal stroke is different from controls, we used diffusion imaging and graph theory to explore structural topology between these populations. Children underwent diffusion and anatomical 3T MRI. Whole-brain tractography was constrained using a brain atlas creating an adjacency matrix containing connectivity values. Graph theory metrics including betweenness centrality, clustering coefficient, and both neighbourhood and hierarchical complexity of sensorimotor nodes were compared to controls. Relationships between these connectivity metrics and validated sensorimotor assessments were explored. Eighty-five participants included 27 with venous stroke (mean age = 11.5 ± 3.7 years), 26 with arterial stroke (mean age = 12.7 ± 4.0 years), and 32 controls (mean age = 13.3 ± 3.6 years). Non-lesioned primary motor (M1), somatosensory (S1) and supplementary motor (SMA) areas demonstrated lower betweenness centrality and higher clustering coefficient in stroke groups. Clustering coefficient of M1, S1, and SMA were inversely associated with clinical motor function. Hemispheric betweenness centrality and clustering coefficient were higher in stroke groups compared to controls. Hierarchical and average neighbourhood complexity across the hemisphere were lower in stroke groups. Developmental plasticity alters the connectivity of key nodes within the sensorimotor network of the non-lesioned hemisphere following perinatal stroke and contributes to clinical disability.

## Introduction

The perinatal timeframe is the most focused period of risk for stroke with the birth prevalence of perinatal stroke approaching 1:1000 births^1^. Perinatal strokes lead to lifelong disability for millions worldwide^1,2^. Three types predominate including arterial ischemic stroke (AIS), encompassing both neonatal arterial ischemic stroke and arterial presumed perinatal ischemic stroke, and periventricular venous infarction (PVI)^1,3^. Males appear to have a higher prevalence than females for neonatal AIS and PVI, but not arterial presumed perinatal ischemic stroke^1^. Although AIS and PVI groups differ in lesion size and location, they share the common complication of hemiparetic cerebral palsy—lifelong weakness to one side of the body^4^.

The injured hemisphere has been a long-standing focus of research, however recent studies have begun to reveal atypical structure and connectivity of the non-lesioned hemisphere in children with perinatal stroke. The altered development of this uninjured tissue may play a major role in determining clinical function^5–8^. Reconstruction of structural connectivity, via tractography using diffusion MRI, combined with mathematical models assessing topology of the human brain via graph theory, may help elucidate the role of the non-lesioned hemisphere in perinatal stroke^5,9–11^. Yet to be identified are the connectivity properties of key nodes within the sensorimotor network of the non-lesioned hemisphere that determine outcome.

Here, we utilize diffusion tractography combined with graph theory to investigate sensorimotor network topology of the non-lesioned hemisphere in children with perinatal stroke and assess relationships with motor function. We hypothesized that the sensorimotor network of the non-lesioned hemisphere reorganizes to prioritize connections between critical structures and that this reorganization is related to motor function.

## Methods

A list of abbreviation can be seen in Supplementary Table 1.

### Participants

Children with perinatal stroke were identified from a large population-based clinical cohort^12^ and a subset of that group was included here. This sample is the same as that reported in a previously published study^5^. Inclusion criteria were: (1) MRI-confirmed unilateral perinatal stroke (AIS or PVI) as determined via a central review by a pediatric neurologist (AK) based on established criteria^13^, (2) symptomatic hemiparetic cerebral palsy (self-described functional limitations by child and parent and Manual Ability Classification System (MACS) score of 1–4)^14^, (3) age 6–19 years, (4) term-birth, and (5) anatomical T1-weighted and diffusion tensor imaging per below. Exclusion criteria were: (1) MRI contraindications, (2) evidence of multifocal, bilateral or diffuse injury at any location in the brain (including subcortical and deep grey structures), (3) unstable epilepsy, (4) poor image quality (venetian blind artifacts in T1 image or < 25 acceptable diffusion volumes confirmed by two neuroimaging scientists (BTC and AJH)), and (5) inability to process neuroimages.

Typically developing controls (TDC) similar in age were recruited from a large control database^15^. Control participants had no history of neurological or neurodevelopmental conditions, were right-handed by self or parent report, and had no MRI contraindications.

All participants provided informed consent/assent or parental consent to participate in this study. This study was approved by the University of Calgary Research Ethics Board and all methods were performed in accordance with the relevant guidelines and regulations.

### Neuroimaging acquisition

All participants completed a standard MRI Neuroplasticity Protocol on a 3 Tesla General Electric MR750w scanner with a 32-channel head coil. High resolution T1-weighted fast spoiled gradient echo brain volume (FSPGR BRAVO) were acquired in the axial plane (voxels = 1 mm isotropic, 166–225 slices, repetition time (TR) = 8.5 ms, echo time (TE) = 3.2 ms, inversion time (TI) = 600 ms, flip angle = 11°, duration =  ~ 6:00). Axially acquired diffusion tensor images were also collected (voxels = 2.5 mm isotropic, 32 non-collinear directions, 60 slices, b-value = 750 s/mm^2^, 3 b = 0 volumes, TR = 11.5 s, TE = 69 ms, duration ~ 6:00).

### Lesion characterization

Using T1-weighted anatomical images, lesions from AIS patients were classified into either proximal or distal M1 involvement, where proximal M1 occlusions had subcortical involvement (i.e., basal ganglia) and distal M1 occlusions did not^13^. Lesion volume was measured using the semi-automatic tracing mechanism within MRIcron^16^. First, a 3-dimensional fill was initiated at the centre of the lesion and dilated from the centre until it reached a change in signal intensity demarcating the lesion boundary. Lesion tracings were then checked manually on the axial, coronal, and sagittal slices and edited to ensure that only lesioned areas were included. Lesion volume (in cc) was then extracted and used in analysis as a potential covariate.

### Image preprocessing

**Figure 1:**
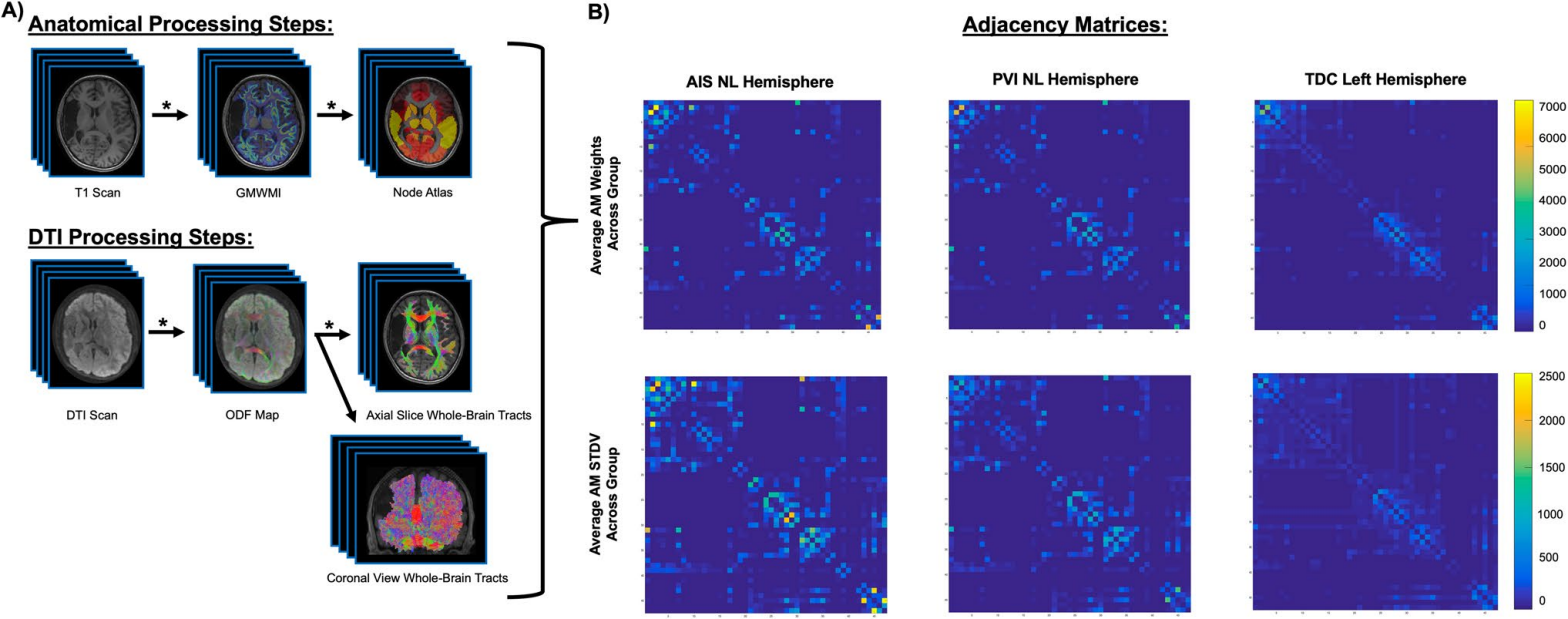
Neuroimage processing steps. (**A**) Anatomical images were segmented based on tissue type and combined to create a gray matter-white matter interface (GMWMI) image. The AAL2 atlas (node atlas) was co-registered into patient diffusion space. For DTI images, eddy currents and small head motion was corrected. ODF maps were then generated and whole-brain tracts were reconstructed (restricted to only white matter using the GMWMI image) and seeded using the co-registered node atlas to generate an undirected 47 × 47 node adjacency matrix containing number of streamlines between node pairs (network weights). Network weights in the non-lesioned hemispheres were compared between groups of children with perinatal stroke (AIS, PVI) and the left hemisphere in controls. Asterisks highlight steps where quality assurance was performed by two authors. (**B**) Group average adjacency matrices for AIS, PVI and TDC participants as well as matrices containing standard deviations to visually illustrate variances between the groups. Images in (**A**) were generated using MRTrix3 (https://www.mrtrix.org/) and images in (**B**) were generated using MATLAB (https://www.mathworks.com/products/matlab.html). *DTI* diffusion tensor image, *ODF* orientation density function, *AIS* arterial ischemic stroke, *PVI* periventricular venous infarction, *TDC* typically developing controls.

Processing steps are identical to our previously published work and are outlined in Fig. 1^5^. Anatomical T1 images underwent preparation for anatomically constrained tractography (ACT)^17^. Due to the heterogenous nature of the size and location of perinatal strokes, multiple software packages were used to optimize the preprocessing and analysis pipelines. T1 scans were parcellated into gray matter, white matter, cerebrospinal fluid, bone, and air using the Statistical Parametric Mapping segmentation tool (https://www.fil.ion.ucl.ac.uk/spm/). To permit reconstructed streamlines to pass through subcortical motor structures (e.g., thalamus), we isolated subcortical structures using FMRIB Software Library (FSL)’s FIRST, then removed these structures from the termination processing within ACT^18^. Once all segmentations were completed, the interface between gray and white matter was generated and this image served as both the initial tractography seed and termination point to constrain reconstructed streamlines to only white matter^17^.

FSL’s FLIRT was used to linearly transform the T1 and respective segmentation masks and volumes into the participant’s diffusion image space. All final co-registered images were quality checked slice-by-slice axially.

Diffusion scans underwent standard pre-processing steps including eddy current and small motion correction by FSL’s eddy_correct. Subsequently, each diffusion volume was quality checked slice-by-slice coronally. Participants that had poor image quality due to artifacts, such as motion or echo planar imaging distortions were excluded from analysis. Whole-brain probabilistic constrained spherical deconvolution tractography using MRTrix3’s tckgen was employed using ACT (angle threshold = 45°; step size = 1 mm; FOD amplitude = 0.05; 1 million streamlines)^17,19^.

Similar transformation steps as above were utilized to transform the automated anatomic labelling 2 atlas (AAL2) to each participant’s diffusion space. In addition, a non-linear transformation using FSL’s FNIRT was used to enhance accuracy of this final coregistration step. ROIs from the warped AAL2 atlases were used to constrain tractography to specific regions.

Quality assurance of image quality and processing steps were assessed slice-by slice axially by two researchers (BTC and AJH) at various steps. These included initial review of the scans before any processing, review following processing of the DTI scan, review of the anatomical segmentation and production of the gray matter white matter interface image, review of the tractography, and finally the review of the atlas parcellation registration to the patient’s brain (Fig. 1, denoted by asterisks).

### Structural matrix processing

A whole brain, undirected 120 × 120 adjacency matrix was generated using MRTrix3’s tck2connectome. The number of streamlines that entered any ROI were used as network weights. Small sub-cerebellar ROIs were subsequently excluded and the non-lesioned hemisphere was isolated leaving a 47 × 47 ROI matrix where the matrix diagonal was set to 0^5^. In controls, streamlines in the left ‘dominant’ hemisphere were used for comparison. The metrics mentioned below were extracted for the whole hemisphere as well as a subset of sensorimotor-related nodes identified using the AAL2 atlas. These nodes included the primary motor cortex (M1), primary somatosensory cortex (S1), supplementary motor area (SMA), thalamus, caudate, putamen, pallidum and one negative control node (inferior occipital gyrus, IOG) to establish functional specificity^20^. Density-based thresholding removed the bottom 25% of weights in each adjacency matrix to exclude spurious reconstructed fibres. Null, or randomized graphs, were generated by creating a randomized 47 × 47 matrix using the Erdös-Renyi algorithm^21^. Based on an average of each group’s sample size, 28 null graphs were generated for comparison.

### Graph theory outcomes

#### General terminology

**Figure 2:**
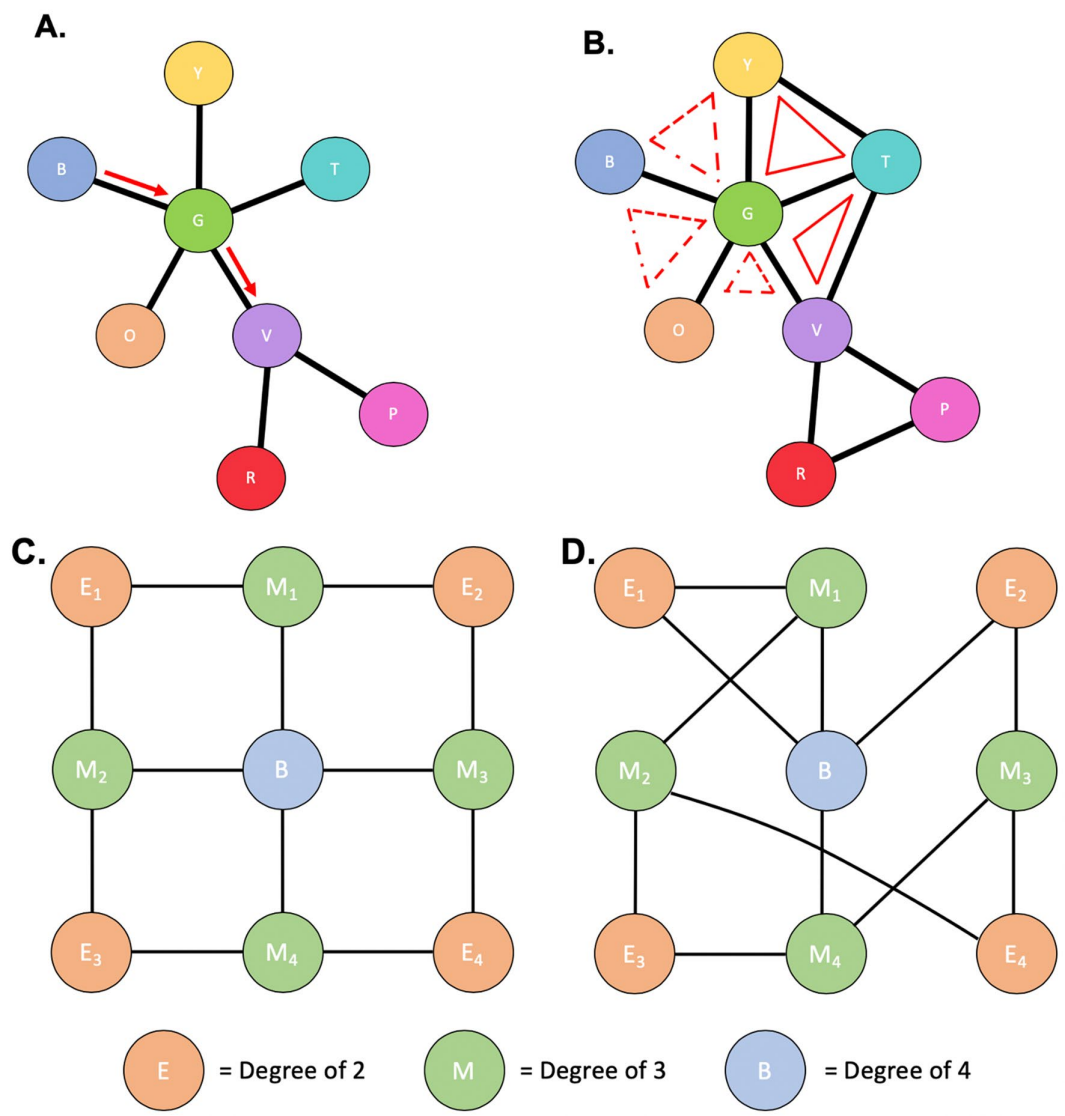
Graph theory application. Figure depicts various topological representations to help explain graph theory concepts. Circles represent a node, whereas the lines represent the edge or weight between each node. In (**A**), the arrow represents the shortest path from node B to node V. In (**B**), the red triangles represent closed triplets, whereas the dashed triangles represent possible triplets.

Graph theory definitions are explained graphically in Fig. 2. A node is defined as an atlas-based region of interest, such as the thalamus or M1. An edge is the connection that joins one node to another. Degree is defined as the number of edges associated with a given node. For example, if a node has 4 edges, it is connected to 4 other nodes and has a degree of 4. A path is defined as a finite sequence of distinct edges and nodes that join together two nodes of interest, such as the red arrows in Fig. 2A. A neighborhood is the set of nodes who are a path length of 1 away from a node of interest.

#### Betweenness centrality

Betweenness centrality assesses how ‘central’ a node is to the network^22^. For example, in Fig. 2A, node G has a high betweenness centrality since any path between nodes O, B, Y, and T all must go through G. Nodes O, B, Y, and T are nodes of degree 1 (only one edge), also called leaf nodes. Thus, no path between any two nodes would pass through them in the network, resulting in a betweenness centrality of 0. Betweenness centrality for a node is defined below, where $\uprho$ is the number of shortest paths between h and j, and ${\uprho }_{\text{hj}}$ is the total number of shortest paths between h and j that pass through node i^22^:

$${\text{b}}_{\text{i}}=\frac{1}{(\text{n}-1)(\text{n}-2)} \sum_{\begin{array}{c}{\text{h}}, {\text{j}}\in {\text{N}}\\ {\text{h}}\ne {\text{j}}, {\text{h}}\ne {\text{i}}, {\text{j}}\ne {\text{i}}\end{array}}\frac{{\uprho }_{\text{hj}}(\text{i})}{{\uprho }_{\text{hj}}}.$$

#### Clustering coefficient

Clustering coefficient assesses a node’s neighbours’ relative connectivity^22^. Specifically, Clustering coefficient evaluates connectivity by determining the number of neighbours which are closed triplets (i.e. a node that is connected to two different nodes that are connected to each other; G-Y-T, for example in Fig. 2B) versus all triplets. The more connected a node’s neighbours are the higher a node’s clustering coefficient is. For example, node G has five neighbours. Two out of those five neighbours have a closed triplet, so G has a lower clustering coefficient compared to node V in which all neighbours contain closed triplets. Clustering coefficient can be defined below, where C^w^ is the weighted clustering coefficient, t_i_^w^ denotes the number of triplets around i, and k being the number of edges that are connected to the vertex i^23^:

$${\text{C}}^{\text{W}}=\frac{1}{\text{n}}{\sum }_{\text{i}\in \text{N}}\frac{2{\text{t}}_{\text{i}}^{\text{w}}}{{\text{k}}_{\text{i}}({\text{k}}_{\text{i}}-1)}.$$

#### Hierarchical complexity

The concept of hierarchy in network topology is often of interest when assessing models through graph representations^24,25^. The brain is a complex system, and as such, models of its topology require an appropriate tool to represent this complexity. To this end, hierarchical complexity has been proposed as a tool which can assess network-wide patterns in connectivity for nodes of the same degree. Hierarchical complexity assesses at any given node how many other nodes that given node is connected to^24^. Here, in a highly ordered system, all nodes would be connected to other nodes with a similar degree. The more ordered a system is, the less complex it is. For example, in Fig. 2C, all nodes with a degree of 2 are connected to nodes of degree 3, meaning that the system is very ordered and has a lower hierarchical complexity. In comparison, in Fig. 2D, nodes with a degree of 2 are connected to nodes with either a degree of either 3 or 4. This system is more complex and in turn has a higher hierarchical complexity. Hierarchical complexity is defined as follows, similar to prior studies^25,26^. Let G = (V,E) represent a graph with given nodes V = {1,…,n} and edges E = {(i,j) : i,j ∈ V}. Also, let $\text{K}=\{{\text{k}}_{1},\dots ,{\text{k}}_{\text{n}}\}$ be the set of degrees of G, $\mathcal{K}$_i_ is the number of edges adjacent to vertex i, with ${\mathcal{K}}_{\text{p}}$ representing the set of nodes of degree p. Then, if ${\text{s}}_{\text{i}}^{\text{p}}$ is the neighbourhood degree sequence ${\text{s}}_{\text{i}}^{\text{p}}=\{{\text{s}}_{\text{i}}^{\text{p}}(1),\dots ,{\text{s}}_{\text{i}}^{\text{p}}(\text{p})\}$ of a node i of degree p, then the hierarchical complexity R is

$$\text{R}=\frac{1}{\mathcal{D}}\sum_{{\mathcal{K}}_{\text{p}}, \left| {\mathcal{K}}_{\text{p}}\right|>1}\frac{1}{\text{p}( \left| {\mathcal{K}}_{\text{p}} \right|-1)}\left(\left.\left.\sum_{\text{j}=\text{i}}^{\text{p}}\left(\sum_{\text{i}\in {\mathcal{K}}_{\text{p}}}({\text{s}}_{\text{i}}^{\text{p}}\left(\text{j}\right)- {\upmu }^{\text{p}}\left(\text{j}\right){)}^{2}\right.\right)\right)\right.,$$

where $\mathcal{D}$ is the total number of distinct degrees in the entire network, and ${\upmu }^{\text{p}}$ is the mean of the jth entries of all p length neighbourhood degree sequences^24,25^.

#### Neighbourhood complexity

Similar to hierarchical complexity, neighbourhood complexity assesses how many nodes is a given node connected to. This metric is calculated at the nodal level, e.g. each node’s unique neighborhood is investigated with respect to their relative complexity. Average neighbourhood complexity is reported here as an average of all of the nodes’ neighbourhood complexities in the hemisphere of interest.

#### Clinical motor function

Children with perinatal stroke completed a battery of evidence-based, validated clinical motor assessments administered by experienced pediatric occupational therapists. The assisting hand assessment (AHA) assesses bimanual upper-extremity function^27^. Children are asked to use their affected hand during a play-based task, leading to a score on each of 22 bimanual hand actions^27^. Total scores are expressed as a logit unit out of 100 with higher scores indicating better performance^27^. The Melbourne assessment (MA) is a unimanual upper-extremity function assessment of the affected side^28^. Sixteen hand/arm functions are assessed, including grasping, reaching, and manipulation. Total scores are out of 100 with higher scores indicating better performance. In the Box and Blocks Test (BBT) a child is asked to quickly and accurately lift one block from one box over a partition and place it in another box as many times as they can within a 60 s span^29^. Tests are completed for both the affected (BBTA) and unaffected hands (BBTU). Total scores represent the number of blocks transferred successfully.

### Statistical analysis

Distribution normality was assessed using the Shapiro–Wilk Test of Normality and subsequent statistical tests were parametric or non-parametric as appropriate.

Differences in age and proportion of sex between the three groups were compared using a Kruskal–Wallis test and Pearson’s Chi-squared test, respectively. Relationships between lesion size, participant age, and all relevant outcomes were investigated using Pearson correlation coefficients (r) or Spearman’s rho (r_s_). Betweenness centrality, clustering coefficient, and neighbourhood complexity was assessed for seven individual sensorimotor-related nodes including M1, S1, SMA, thalamus, caudate, putamen, pallidum and one negative control node (IOG). The above metrics (BC, CC, and NC) and hierarchical complexity were then assessed the non-lesioned hemisphere in its entirety (i.e. encompassing an average of all 47 nodes) between groups using either an analysis of variance or a Kruskal–Wallis test. Bonferroni correction for multiple comparisons was employed. Relationships between clinical motor assessments and graph theory outcomes, both at the nodal and hemispheric levels, were assessed using either Pearson correlations (r) or Spearman correlations (r_s_), depending on normality. Results for the relationships between hemispheric averages and clinical motor outcomes were assessed both as a pooled group (i.e., AIS and PVI combined) and separated. All network metrics mentioned above were compared to metrics generated from a null, randomized graph between each group that underwent similar density-based thresholding. Cohen’s d was calculated for comparisons that were normally distributed to assess effect sizes. Post-hoc power calculations were performed using G*Power^30^ with an alpha-level of 0.05, an effect size of 1.0 (a conservative estimate based on current observed effect sizes), and the sample size of the two smallest groups in our study.

## Results

### Population

The initial sample consisted of 90 participants. Five were excluded due to poor image quality (n = 3) or inability to process data due to large lesions (n = 2). The final sample consisted of 85 participants, including 27 with PVI (63% male; mean age = 11.5 ± 3.7 years; range 6.6–19.7 years), 26 with AIS (58% male; mean age = 12.7 ± 4.0 years; range 6.6–19.0), and 32 TDC (53% male; mean age = 13.3 ± 3.6 years; range 6.4–19.0 years). Median age and sex proportions did not differ between groups (H_(2,82)_ = 4.307, p = 0.366; $\upchi$^2^_(2)_ = 0.582, p = 0.748). For AIS patients, 62% had proximal M1 occlusions (basal ganglia involvement) and the remaining 38% had distal M1 occlusions (basal ganglia spared). Age, sex, and lesion size were not associated with any graph theory outcomes (all p > 0.05, all r ≤ 0.112).

A subset of the perinatal stroke population completed motor assessments. Due to small samples within each stroke subtype, participants were combined into one group to maximize statistical power. Forty-nine participants completed the AHA (mean logit score = 61.9 ± 18.2; AIS N = 26, mean logit score = 56.1 ± 18.7; PVI N = 23, mean logit score = 68.4 ± 15.7). Thirty-four completed the MA (mean = 76.7 ± 20.6; AIS N = 20, mean = 68.8 ± 22.4; PVI N = 14, mean = 88.0 ± 10.6). Forty-nine completed the BBT where the BBTA scores (mean = 29.3 ± 16.4) were lower than BBTU scores (mean = 46.5 ± 15.7; t_(46)_ = − 4.808; p < 0.001, d = 1.07). For AIS patients, 26 patients completed the BBT (mean BBTA = 23.0 ± 16.6; mean BBTU = 44.0 ± 14.5), whereas 23 PVI patients completed the BBT (mean BBTA = 36.3 ± 13.4; mean BBTU = 49.0 ± 16.8). 93% of participants completed at least two of the three motor assessments.

### Sensorimotor network nodal metrics between groups

#### Primary motor cortex (M1)

**Figure 3:**
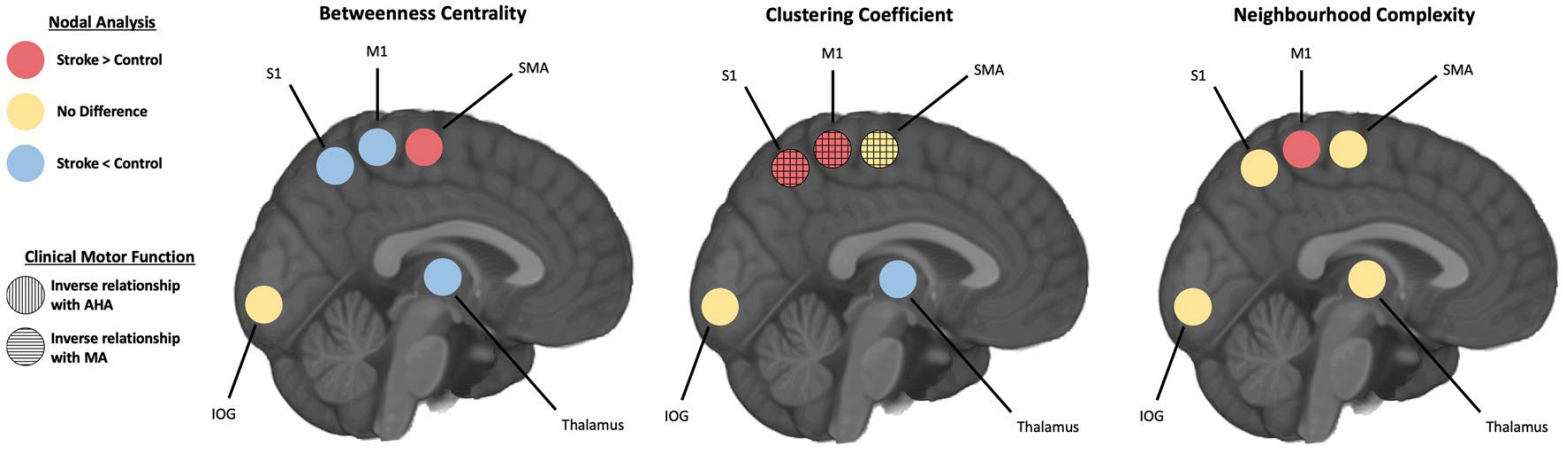
Sensorimotor network node analysis. Sensorimotor node differences between the dominant hemisphere of controls to the non-lesioned, intact hemisphere of both stroke groups are shown. For the simplicity of the diagram, both arterial and venous strokes were combined as they displayed no differences from each other in any of the nodes. Red circles represent the nodes where both stroke groups showed higher values compared to controls, yellow representing no difference, and blue representing nodes of lower values compared to controls. Vertical lines represent an inverse relationship between the respective graph theory metric at the node of interest and the AHA. Horizontal lines represent the same inverse relationship with the node, but in relationship to MA instead. *S1* primary sensory cortex, *M1* primary motor cortex, *SMA* supplementary motor area, *IOG* inferior occipital gyrus, *AHA* assisting hand assessment, *MA* Melbourne assessment.

Group differences among sensorimotor network metrics as well as relationships with function are portrayed in Fig. 3. Betweenness centrality of TDC M1 (309 ± 120) was higher (H_(2,82)_ = 52.63; p < 0.001) than AIS (68 ± 65; p < 0.001), and PVI (62 ± 55; p = 0.003). Clustering coefficient of TDC M1 (138 ± 23) was lower (H_(2,82)_ = 59.59; p < 0.001) than AIS (266 ± 75; p < 0.001) and PVI (226 ± 33, p < 0.001). Neighbourhood complexity of TDC M1 (0.018 ± 0.009) was lower (H_(2,82)_ = 7.92; p = 0.019) than AIS (0.053 ± 0.050; p = 0.028) and PVI (0.058 ± 0.050; p = 0.020).

#### Primary somatosensory cortex (S1)

Consistent with M1, betweenness centrality of TDC S1 (295 ± 137) was significantly higher (H_(2,82)_ = 31.01; p < 0.001) compared to AIS (137 ± 110; p < 0.001) and PVI (112 ± 87; p < 0.001). Clustering coefficient was lower (H_(2,82)_ = 44.26; p < 0.001) in TDC S1 (121 ± 24) compared to both AIS (211 ± 58; p < 0.001) and PVI (179 ± 36; p < 0.001). Neighbourhood complexity showed no differences (p = 0.13).

#### Supplementary motor area (SMA)

Betweenness centrality of TDC SMA (18.1 ± 17.2) was lower (H_(2,82)_ = 26.28; p < 0.001) than AIS (87.8 ± 65.5; p < 0.001) and PVI (72.1 ± 66.7; p < 0.001). No other significant differences were found (all p > 0.19).

#### Subcortical gray motor areas (thalamus, caudate, putamen, pallidum)

Clustering coefficient of TDC thalamus (268 ± 78) was higher (H_(2,82)_ = 19.3; p < 0.001) than both AIS (79 ± 26; p = 0.009) and PVI (58 ± 18; p < 0.001). No other graph theory metrics showed group differences for the other subcortical areas (all p > 0.24).

#### Inferior occipital gyrus (IOG)

No differences were observed between any of the groups for any metrics in the IOG (p = 0.42).

#### Motor network metrics and clinical motor function

M1 clustering coefficient was inversely related to both the AHA (r_s_ =  − 0.329, p = 0.021) and the MA (r_s_ =  − 0.415, p = 0.015). S1 clustering coefficient was inversely associated with both the AHA (r_s_ =  − 0.446, p < 0.001) and MA (r_s_ =  − 0.502, p = 0.003). SMA clustering coefficient was inversely related to both AHA (r_s_ =  − 0.333, p = 0.019) and MA (r_s_ =  − 0.359, p = 0.037). No significant relationships were found between any of the graph theory metrics for subcortical gray motor areas and clinical motor function (all p > 0.47). There were no apparent relationships with the graph theory metrics for IOG and any motor function assessments (all p > 0.57). There were no significant associations between motor network metrics and the BBT (minimum p = 0.21).

### Hemispheric network metrics between groups

**Figure 4:**
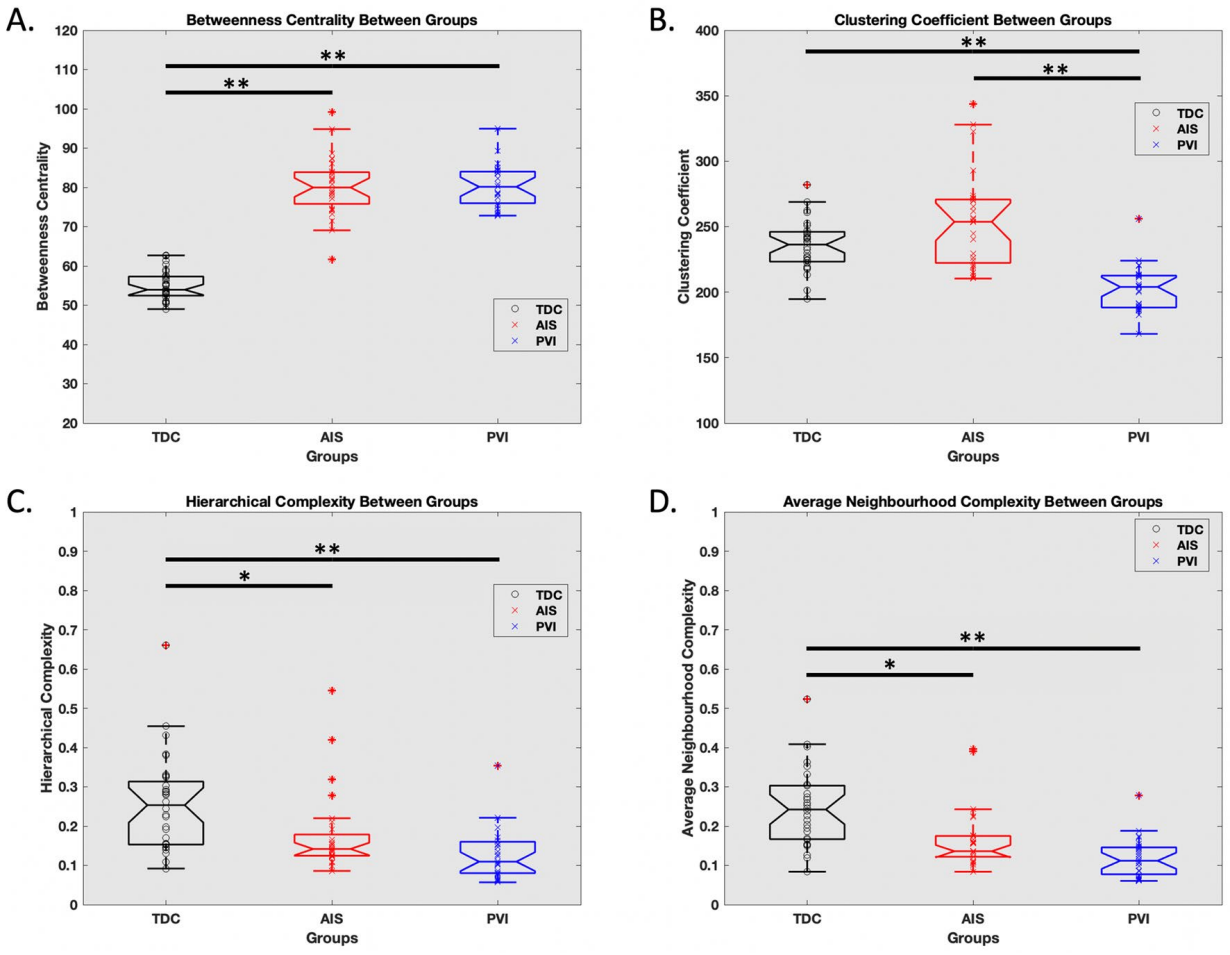
Hemispheric network metrics between group comparisons. (**A**) Betweenness centrality was higher in AIS and PVI compared to TDC. (**B**) Clustering coefficient was higher in TDC and AIS compared to PVI. Hierarchical complexity, (**C**) average neighbourhood complexity, (**D**) were higher in TDC compared to AIS and PVI. **p < 0.001, *p < 0.01.

Betweenness centrality was higher in both AIS (80.3 ± 7.6, p < 0.001, d = 4.27) and PVI (80.2 ± 5.3, p < 0.001, d = 5.58) compared to TDC (54.9 ± 3.6; F_(2,82)_ = 202.8; p < 0.001; Fig. 4A). Clustering coefficient in PVI (202 ± 17) was lower (H_(2,82)_ = 41.61; p < 0.001; Fig. 4B) compared to AIS (253 ± 36; p < 0.001) and TDC (235 ± 19; p < 0.001). Hierarchical complexity was higher (H_(2,82)_ = 25.27; p < 0.001; Fig. 4C) in TDC (0.256 ± 0.120) compared to PVI (0.128 ± 0.065; p < 0.001) and AIS (0.178 ± 0.102; p = 0.008). Average neighbourhood complexity was higher (H_(2,82)_ = 33.27; p < 0.001; Fig. 4D) in TDC (0.248 ± 0.097) compared to PVI (0.119 ± 0.049; p < 0.001) and AIS (0.166 ± 0.076; p = 0.003). All metrics for each group differed compared to the null graphs (all p < 0.001).

### Hemispheric network metrics and clinical motor function

**Figure 5:**
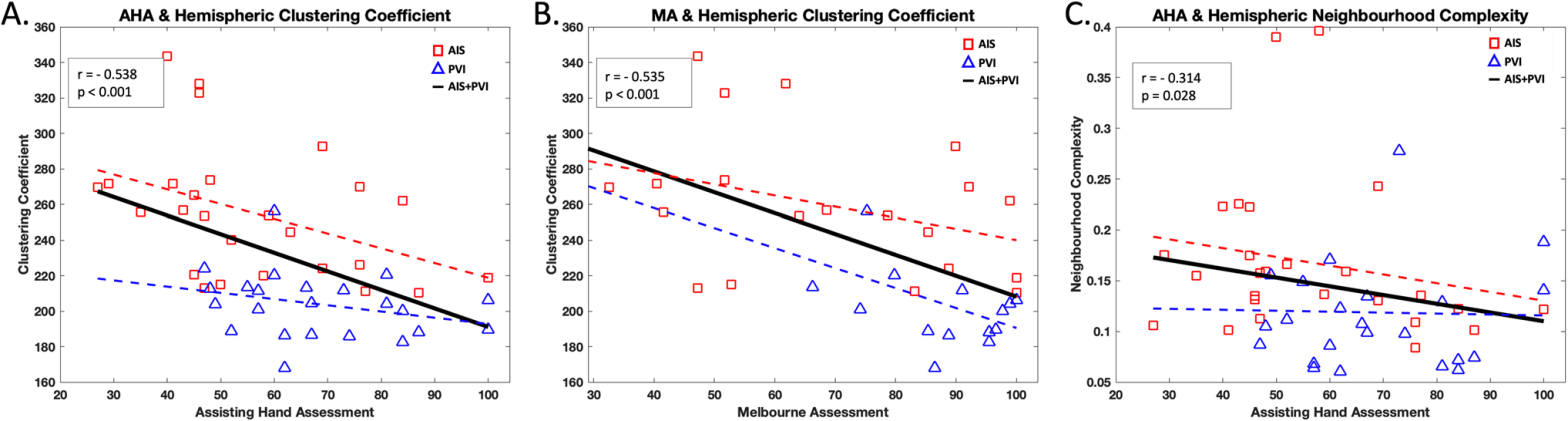
Hemispheric graph theory metrics and clinical motor function. (**A**) Hemispheric clustering coefficient was inversely related to AHA (**A**) and MA (**B**). The average neighbourhood complexity across the hemisphere was also inversely related to AHA (**C**). *AHA* assisting hand assessment, *MA* Melbourne assessment, *AIS* arterial ischemic stroke, *PVI* periventricular venous infarction.

Pooled clustering coefficient was inversely related to both the AHA (r_s_ =  − 0.538; p < 0.001; Fig. 5A) and MA (r_s_ =  − 0.535; p < 0.001; Fig. 5B). AIS average clustering coefficient was inversely related to AHA (r_s_ = − 0.485; p = 0.008; Fig. 5A) and MA (r_s_ =  − 0.325; p = 0.033; Fig. 5B). PVI average clustering coefficient was inversely related to AHA (r_s_ =  − 0.305; p = 0.031; Fig. 5A) and MA (r_s_ =  − 0.504; p = 0.002; Fig. 5B). Pooled average neighbourhood complexity was inversely related to AHA (r_s_ =  − 0.314; p = 0.028; Fig. 5C). AIS average neighbourhood complexity was inversely associated with AHA (r_s_ =  − 0.301; p = 0.039; Fig. 5C), but PVI was not. Betweenness centrality and hierarchical complexity showed no significant relationships with motor function for either pooled or separated analyses (minumum p > 0.06). There were no significant associations between hemispheric outcomes and the BBT with groups pooled or separated (minimum p = 0.17).

### Post hoc power analysis

Statistical power was estimated as 94.6% for the AIS vs TDC group comparison for hemispheric clustering coefficient, using an alpha-level of 0.05, sample sizes of n = 26 and n = 27, and a relatively conservative effect size of d = 1.0. Power values for additional dependent variables were similar or higher. Our actual effect sizes reported above were much higher than this (d =  ~ 4.5), thus we feel confident that the likelihood of reporting a false positive (or false negative) was low.

## Discussion

Evaluating both motor-related and hemispheric nodes, we used graph theory metrics to reveal consistent moderate differences in structural connectivity of the non-lesioned hemisphere in children with perinatal stroke compared to peers. Modest relationships with clinical motor function were found, both at the sensorimotor network and hemispheric levels. Using graph theory to understand clinical function is a promising tool to better provide insights into how connections throughout the brain can develop in the face of early unilateral injuries.

Graph-theory metrics in sensorimotor-related areas of the non-lesioned hemisphere provided functionally specific insight. Key sensorimotor areas of M1, S1, and SMA showed consistent differences compared to TDC, along with significant relations to clinical measures. Both non-lesioned M1 and S1 of stroke participants demonstrated a lower betweenness centrality and a higher clustering coefficient compared to TDC. This suggests that these nodes may be similar to ‘leaf’ nodes (e.g., node O in Fig. 2B), where they occupy more terminal positions in the network, either describing a position at the start or end of a path. We also demonstrated that the more connected M1 and S1 neighbors were (e.g., node G in Fig. 2B), as determined by the clustering coefficient, the poorer was the motor function in the group of children with perinatal stroke. Higher network structural connectivity as measured here by clustering coefficient may reflect compensatory inclusion of additional functions in the non-lesioned hemisphere from the lesioned possibly leading to crowding, and less central nodes, resulting in poorer function. This suggestion could be investigated more thoroughly using motor task fMRI to localize specific function.

The significant relationships between M1/S1 metrics and clinical motor function speak to the importance of the potential neuroplastic reorganization of these areas following perinatal stroke. Preclinical studies have demonstrated the ability of the brain to structurally adapt to early injury, where typical contralateral projections from the corticospinal tract can remain bilateral from birth, allowing the non-lesioned hemisphere to potentially control the paretic limb^8,31^. This principle has been confirmed by multiple neurophysiological methods using transcranial magnetic stimulation to stimulate the non-lesioned corticospinal tract, characterizing these ipsilateral connections and their relevance to hand function in children with perinatal stroke^32,33^. The confirmation of such developmental neuroplastic mechanisms supported our hypothesis of increased neighbouring connectivity to support motor function. That M1 of the non-lesioned hemisphere contained a higher clustering coefficient compared to controls may further support our hypothesis that neighbouring connectivity patterns surrounding this critical motor-related node are relevant to motor function.

The non-lesioned hemisphere in its entirety showed differences compared to the dominant hemisphere in controls across all metrics, suggesting broad developmental differences occur after perinatal stroke. These metrics, based on averages across all nodes in the network, provide converging evidence with our prior study investigating different hemispheric connectivity measures that topological outcomes differ in children with perinatal stroke compared to controls^5^. In the non-lesioned hemisphere, betweenness centrality, a measure of how ‘central’ a node is to the network, appeared to be higher in stroke populations compared to controls, suggesting that nodes may be more centralized after stroke. As such, the non-lesioned hemisphere may contain more ‘hub-like’ topology with some regions having greater bearing for connecting the white-matter tracts across the brain. In TDC, the development of structural connectivity trends from ‘global’ to ‘local’ with activity-dependent elimination of excess synapses^10^. However, an early insult such as perinatal stroke may reduce this normal pruning process, leading to higher betweenness centrality in the non-lesioned hemisphere. This finding is consistent with previously published work revealing a more globally efficient, or highly connected, network of the non-lesioned hemisphere compared to the dominant hemisphere of controls^5^.

Hierarchical and neighbourhood complexities were higher in controls than stroke for the entire hemisphere, suggesting that stroke populations may have more ordered networks. This suggests that across the network, regions with similar white matter structural connectivity are more likely to be similarly connected, rather than complexly, such as the node E in Fig. 2C where all E nodes have a degree of two and all connect to nodes of a degree of 3. While ordered networks may instinctively be thought to be advantageous, recent research has shown that more complex neurological networks are associated with the evolution of brain development across species along the phylogenetic scale^34^. That both stroke groups had a less complex network in the non-lesioned hemisphere compared to the dominant hemisphere in controls suggests a differing developmental trajectory following early injury to the opposite hemisphere. Whether this differing trajectory is a relative delay or arrest, a shift in timing, or a prolongation of development is unclear and could be resolved with longitudinal studies. Group differences in network complexity also suggest that more typical brain structure (as seen in TDC) may be associated with more complex networks. It is imperative to explore developmental trajectories in a large typically developing cohort for a more comprehensive understanding of normal topological development so that we can define deviations from that trajectory in perinatal stroke and other neurological diseases. Finally, while group differences were present in our sample for both hierarchical and neighbourhood complexities at both the hemispheric and nodal levels there was a lack of association with function. This suggests that the complexity of topology may not be a strong predictor of motor function but rather reflects how connectivity patterns may be altered following an early brain injury. Future studies could examine how hierarchical and neighbourhood complexities relate to higher-level cognitive processing to elucidate how these complexities in network structure are associated with complexities in cognitive processes.

Three hemispheric graph theory metrics had significant relationships with clinical motor function. Clustering coefficient was negatively correlated with the AHA and MA tests and average neighbourhood complexity was negatively associated with the AHA. Compared to the TDC group, who had higher hemispheric clustering and neighbourhood complexity than stroke, we would expect to have seen the inverse relationship where a higher clustering coefficient and neighbourhood complexity would be associated with better clinical motor function. As these motor function tests require very specific neurological correlates to function, it is difficult to speculate why we saw such negative associations but their consistent presence across multiple outcomes suggests converging evidence. We would also not assume that such simple relationships would explain what must be complex relationships between these many factors. Perhaps the additional investigation of functional connectome topology using functional MRI would provide a unique perspective to the connectivity patterns revealed by the hemispheric clustering and neighbourhood complexity.

Interestingly, when comparing the results from the hemispheric versus nodal metrics, we observed some opposing findings (i.e. hemispheric clustering coefficient lower in the stroke participants compared to controls, but higher when investigating M1 alone). This apparent higher clustering coefficient in M1 may be due to greater connectivity of the surrounding neighbours of M1, suggesting potential neuroplastic compensation leading to control of both the paretic and non-paretic hand. In TDC populations, normal pruning of excess synapses is based on activity-dependent contralateral control of the body. After perinatal stroke, activity-dependent pruning may result in bilateral control of the extremities in M1 of the non-lesioned hemisphere. Our recent task-based functional MRI study potentially confirms this hypothesis, where control of the ipsilesional hand activated areas were found to be remote from where M1 typically is located^35^.

Another possibility may relate to the relative maturation of different systems at the time of injury; the motor system being partially developed while most other major networks are yet to be activated. This discrepancy might explain these contrasting metrics between the motor network as compared to the entire hemisphere. Also, as the hemispheric metric takes an average of all of the nodes of the hemisphere, it may be less functionally-specific compared to the nodal assessment of M1, which would presumably have a more defined relationship with motor function. This also adds an argument for the importance of how hemispheric results may obscure node- or network-specific outcomes and how paramount it is to describe both hemispheric and nodal outcomes. We also observed differential outcomes in hemispheric clustering coefficient according to stroke type where AIS had higher clustering and PVI had lower clustering compared to TDC. While lesions in AIS patients may be more heterogenous compared to PVI, which only involve periventricular areas, structural connectivity reorganization in children with AIS may involve different networks and in turn lead to increased clustering between networks. Whether these are attributable to differences in the size, location, timing of injury, or some combination of factors that may distinguish AIS from PVI is unknown.

We did not see any relationships between lesion size and any graph theory metric. Since we only investigated the non-lesioned hemisphere, perhaps lesion size does not influence network topological patterns in a direct way. Similar to our previous findings that thalamic volume following perinatal stroke was not related to lesion size, it is potentially the lesion location rather than lesion size that may more directly affect motor function^6^. We also did not see any associations between the BBT and nodal or hemispheric graph theory metrics. This apparent lack of association may be because the BBT is a simpler, single-task unimanual motor assessment in contrast to the AHA and MA which are larger test batteries composed of multiple complex tasks. Possibly, more complex bilateral tasks tap multiple areas of the wider motor network and differences are more readily detectible via graph theory metrics manifesting as functional correlations compared to simple unimanual tasks such as BBT. Such findings support reconsideration of how commonly used but simple factors may have limited utility in predictive models of perinatal stroke outcome. Another example may be sex where males are well known to have a higher prevalence for NAIS (Dunbar, 2020) but we observed no sex-specific effects on network outcomes in the nonlesioned hemisphere.

Surprisingly, we did not see any relationships of motor function with subcortical gray matter nodes. Given the central role of the basal ganglia and thalamus in motor circuitry and the close relationship with outcomes after stroke^13^, this finding is somewhat unexpected and the role of these structures in the non-lesioned hemisphere networks remains poorly understood. Our anatomically constrained tractography methods were fairly stringent, thus some reconstructed fibres may have been truncated when passing through narrow subcortical areas. Further, lower signal quality in diffusion MRI near the inferior portions of the brain may have caused subcortical areas to have lower FOD amplitudes, thus terminating tractography streamlines. Particularly surprisingly was that we found no difference or relationship with motor function when examining the structural connectivity of the non-lesioned thalamus. Previous volumetric studies have shown that the non-lesioned thalamus may be larger in size compared to controls and that the degree of size difference was associated with clinical disability^6^. The thalamus is a highly complex and connected structure and more in-depth investigation potentially using tractography to parcellate the thalamus into functionally-specific nuclei may provide further insight.

While there are obvious differences in measures of structural and functional connectivity, the overarching theme that connectivity may be associated with function is shared. We have consistently found in this perinatal stroke population, altered functional and structural connectivity in sensorimotor networks of both hemispheres (and between hemispheres) with many metrics showing strong associations with sensorimotor function^36–40^. The current findings that complex differences in sensorimotor network topology (via graph theory metrics) are also associated with function add additional evidence especially given that findings appear to be specific to the same sensorimotor network nodes as in prior studies. How these stories all come together is an ongoing avenue for future endeavours that multiple imaging modalities and multiple levels of analysis (i.e., measurement of “direct” functional and structural connectivity versus higher brain/hemispheric-level network topologies) will ideally help with, but at the present time, is unclear. The finding that many such metrics are clinically relevant suggests it is important to pursue if attempts to improve function in kids with hemiparetic cerebral palsy are to be realized.

Our study has limitations. The sample we chose included only children with perinatal stroke who could receive an MRI and had some voluntary hand movement, excluding those with the most severe disabilities (i.e., MACS score of 5). Our population was between the ages of 6–19 years to support adherence with scanning protocols and outcome measures, but we may be missing key neuroplastic developmental stages occurring earlier (and we did not observe relationships between any metric and age). We were not able to include the cerebellum, a key structure in the motor network. The diffusion MRI acquisition did not have a large enough field of view to include both M1 and the cerebellum in a portion of our study (~ 30%). The motor function analysis could not be stratified into different subtypes of stroke due to the sample size. The motor assessments used in the current study only investigate upper extremity function and inclusion of measures of lower extremity function as well as other domains such as language, cognition, and more may have been additionally informative. Future studies should collect functional assessments of all children to improve the power of the study, including assessments that are appropriate for testing function in both controls and those with hemiparesis for direct comparison. Comorbid diagnoses of epilepsy and attention deficit hyperactivity disorder occur in perinatal stroke, but we did not include these as covariates since this information was not collected. We do note that performance on motor function testing may have been affected by the presence of these comorbidities and the medication used to treat them. Finally, we only looked at very specific graph metrics while many others may be relevant or insightful. Instead, we attempted to ‘capture’ certain aspects of the network through these metrics such as how central a node is to the network, the connectivity of neighbours, and how complex connections are across the network.

## Conclusion

Graph theory may be a useful tool in understanding the connectivity of the non-lesioned hemisphere in children with perinatal stroke. The structural connectivity of key nodes in the sensorimotor network of the nonlesioned hemisphere is altered after perinatal stroke and related to clinical function. Nodal analysis of such specific networks adds to global evaluations of structural connectivity in the non-lesioned hemisphere.

## Supplementary Information

Supplementary Table 1.

## Data Availability

All neuroimaging data in the present study will be deidentified and made available to qualified researchers upon a reasonable request.
