# Supplementary material for: Structural connectivity of the sensorimotor network within the non-lesioned hemisphere of children with perinatal stroke
Source: Sci Rep. 2022 Mar 9;12:3866. doi: 10.1038/s41598-022-07863-4 (PMC8907195; doi:10.1038/s41598-022-07863-4)
Supplement: Supplementary file 1 — Supplementary Table 1. [file 41598_2022_7863_MOESM1_ESM.docx]

**Supplementary Material**

**Supplementary Table 1**

| **Abbreviation** | **Full Description** |
| --- | --- |
| AHA | Assisting Hand Assessment |
| AIS | Arterial ischemic stroke |
| APPIS | Arterial presumed perinatal ischemic stroke |
| ACT | Anatomically constrained tractography |
| BBT | Box and Blocks Test |
| DTI | Diffusion tensor imaging |
| FSL | FMRIB Software Library |
| FSPGR- BRAVO | Fast spoiled gradient echo brain volumes |
| IOG | Inferior occipital gyrus |
| M1 | Primary motor cortex; OR M1 artery |
| MA | Melbourne Assessment |
| MACS | Manual Ability Classification System |
| MRI | Magnetic resonance imaging |
| NAIS | Neonatal arterial ischemic stroke |
| PVI | Periventricular venous infarction |
| S1 | Primary somatosensory cortex |
| SMA | Supplementary motor area |
| TDC | Typically developing controls |
| TE | Echo time |
| TI | Inversion time |
| TR | Repetition time |

Supplementary Table 1 reveals all abbreviations used throughout the manuscript.

**Figure Generation**

Figure 2 and figure 3 were created using Microsoft Powerpoint (<https://www.microsoft.com/en-us/microsoft-365/powerpoint>).
